# Supplementary figures and images for: Malaria Risk Drivers in the Brazilian Amazon: Land Use—Land Cover Interactions and Biological Diversity
Source: Int J Environ Res Public Health. 2023 Aug 1;20(15):6497. doi: 10.3390/ijerph20156497 (PMC10419050; doi:10.3390/ijerph20156497)

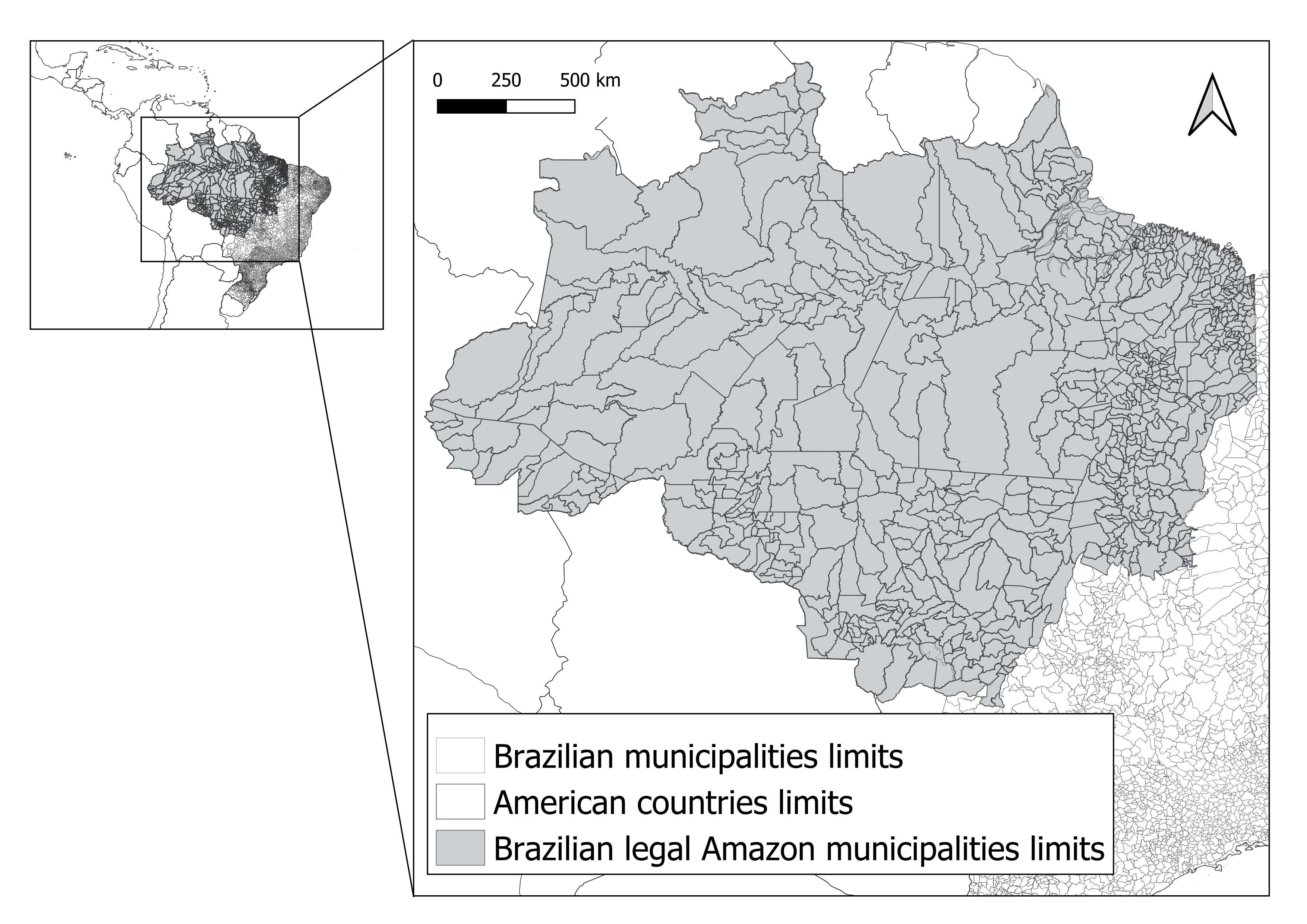

Supplement: Supplementary file 1 [file ijerph-20-06497-s001.zip › Figure S1.png]

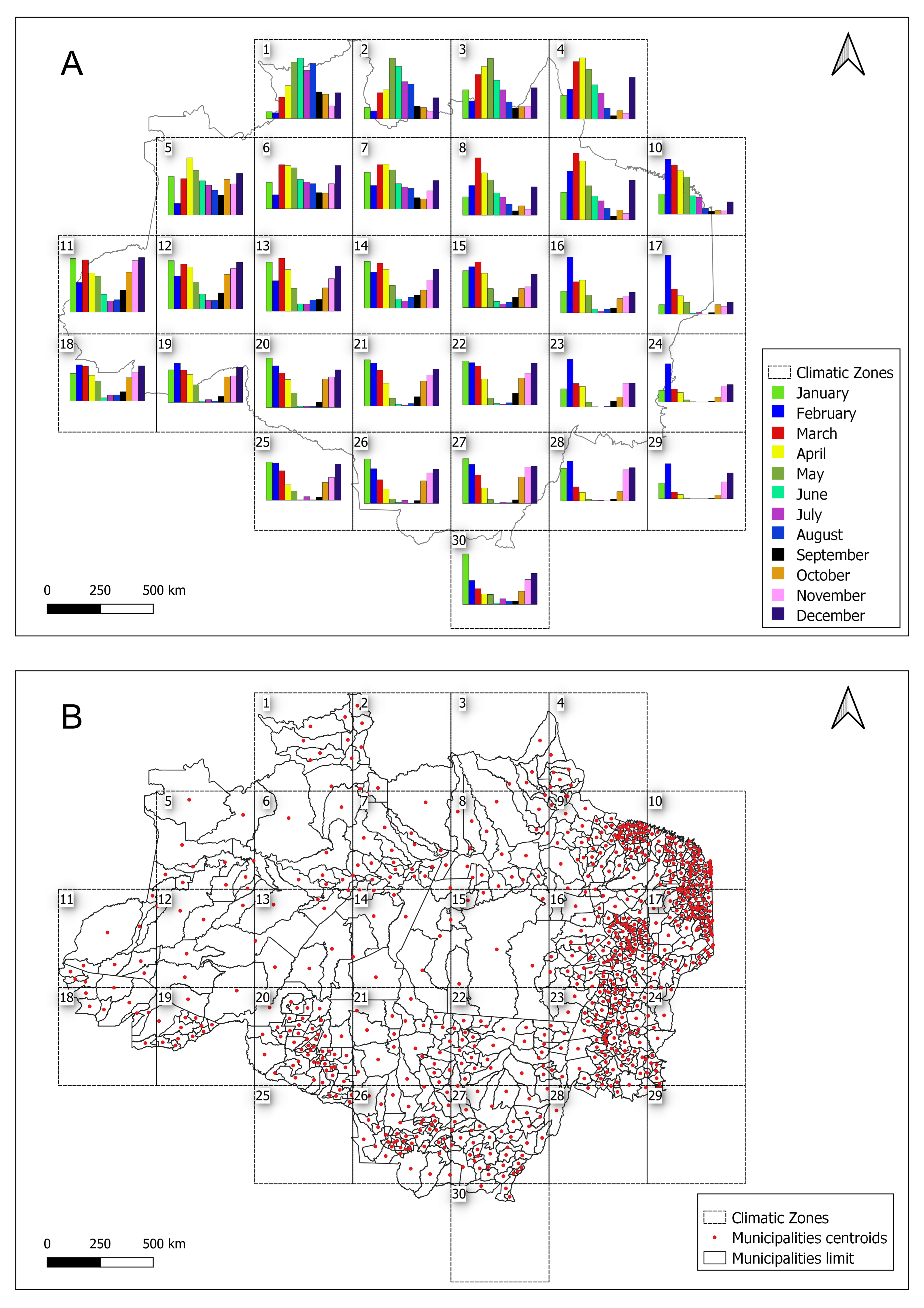

Supplement: Supplementary file 1 [file ijerph-20-06497-s001.zip › Figure S2.jpg]

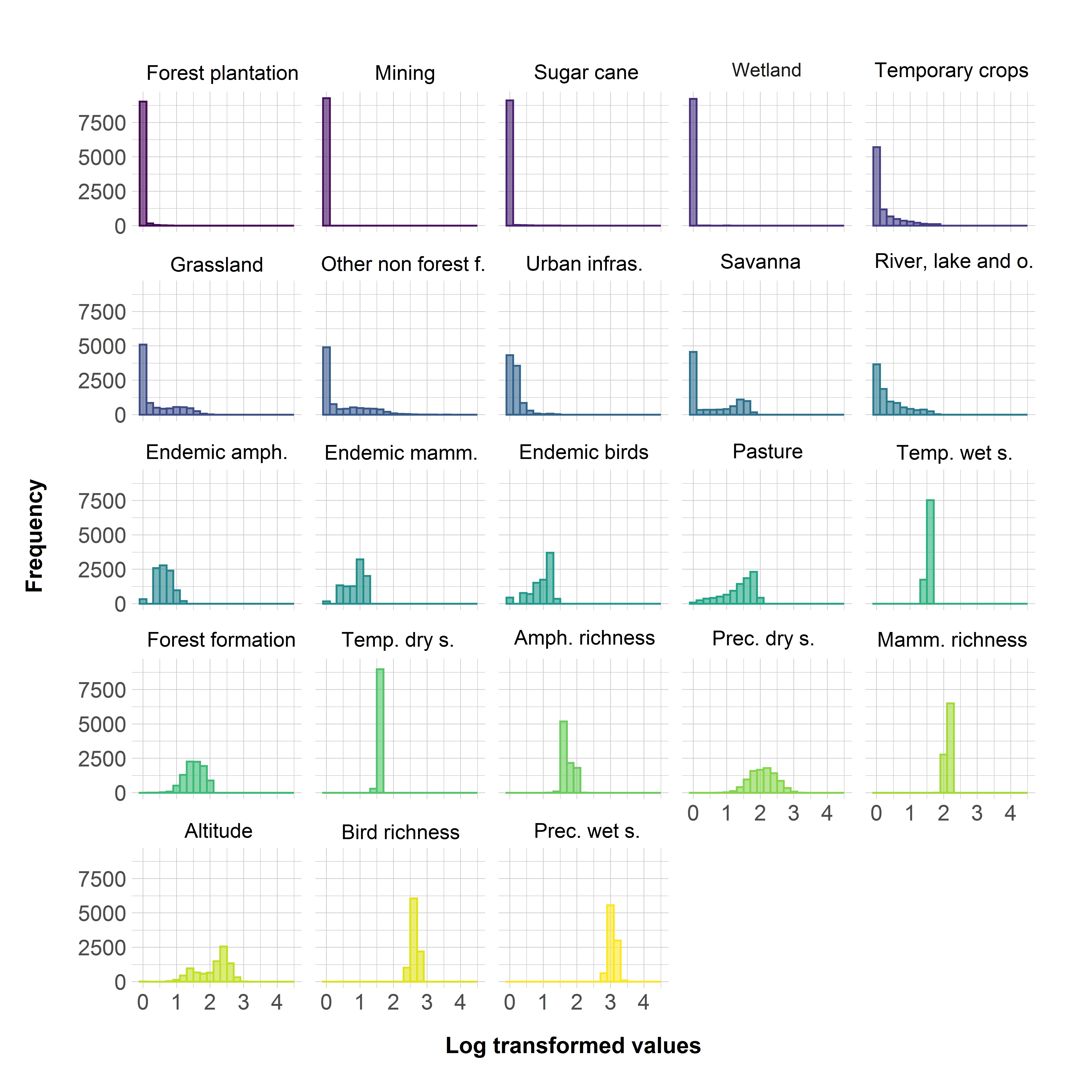

Supplement: Supplementary file 1 [file ijerph-20-06497-s001.zip › Figure S3.jpeg]

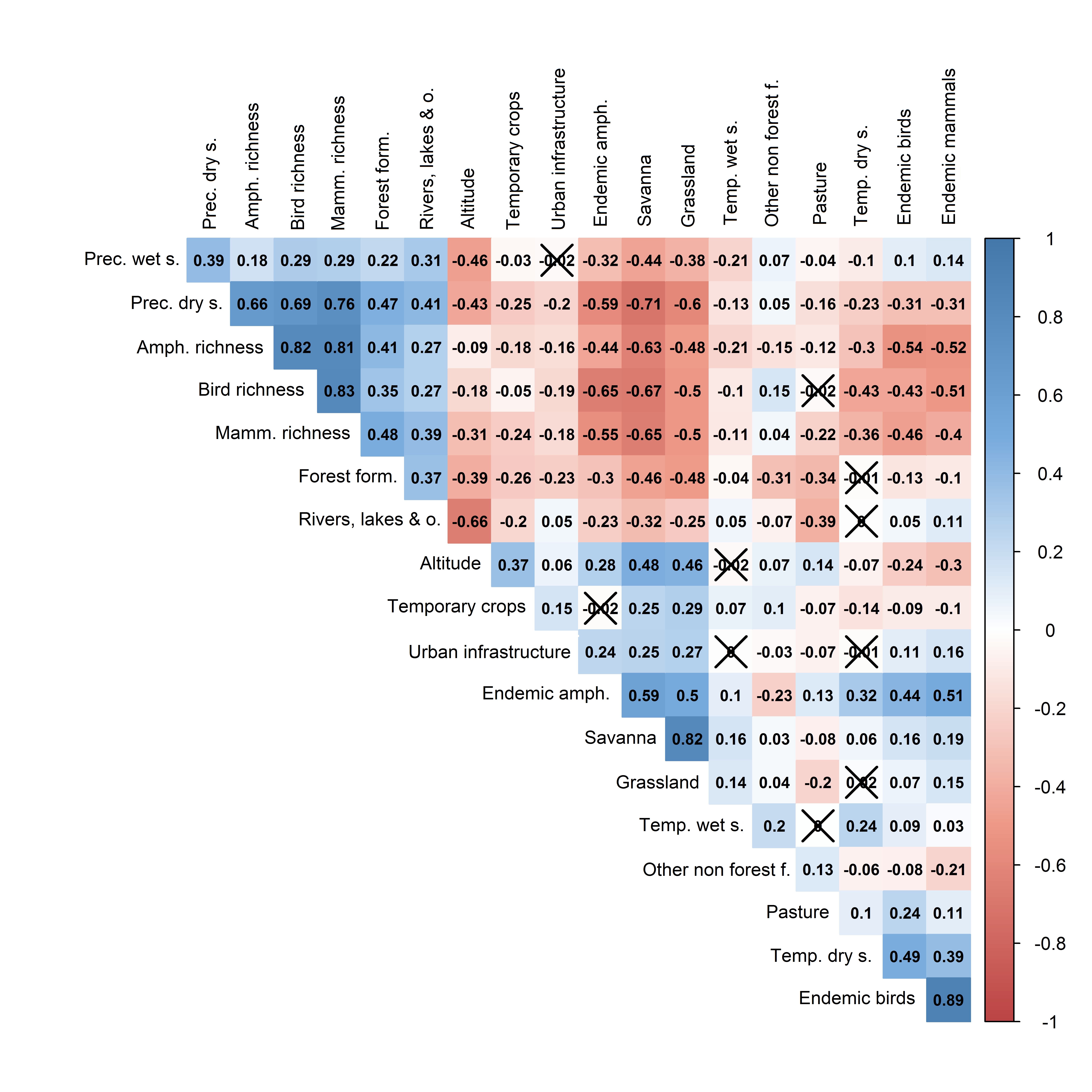

Supplement: Supplementary file 1 [file ijerph-20-06497-s001.zip › Figure S4.jpeg]

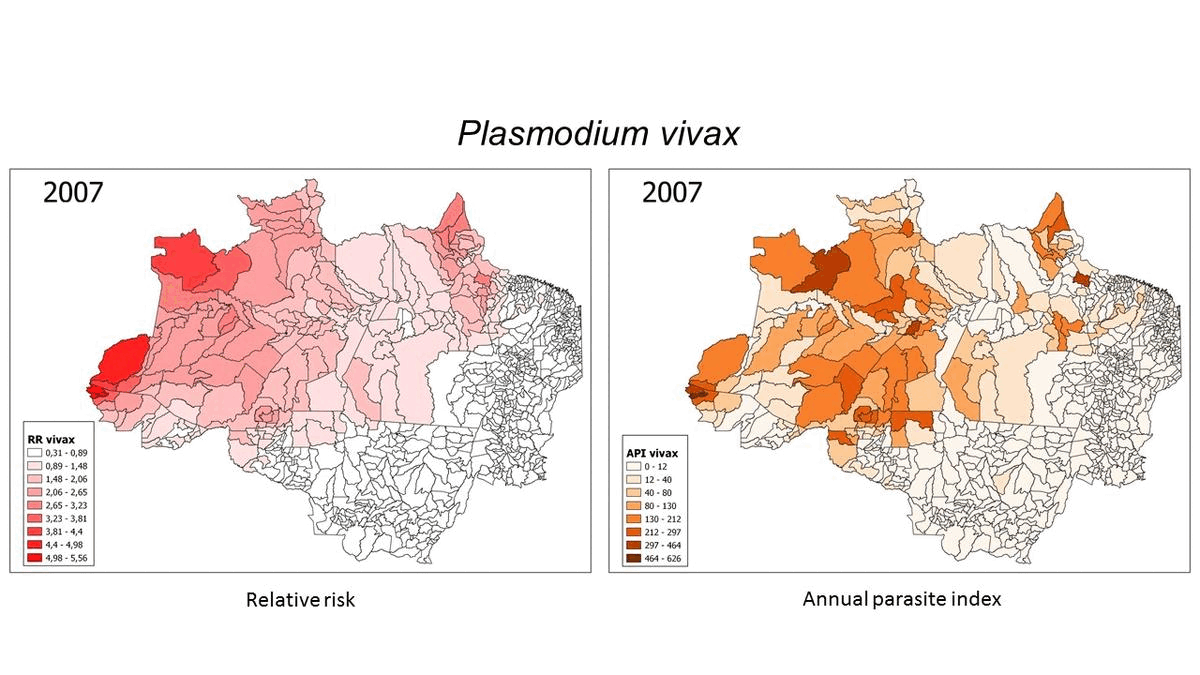

Supplement: Supplementary file 1 [file ijerph-20-06497-s001.zip › Figure S5.gif]

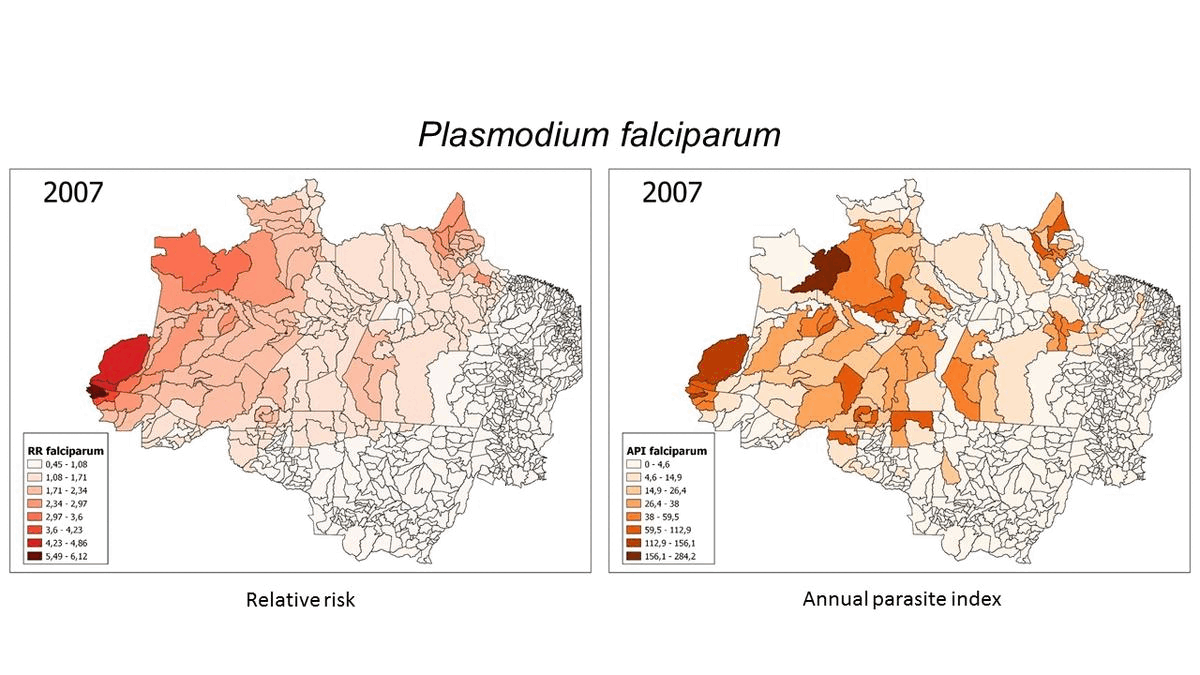

Supplement: Supplementary file 1 [file ijerph-20-06497-s001.zip › Figure S6.gif]

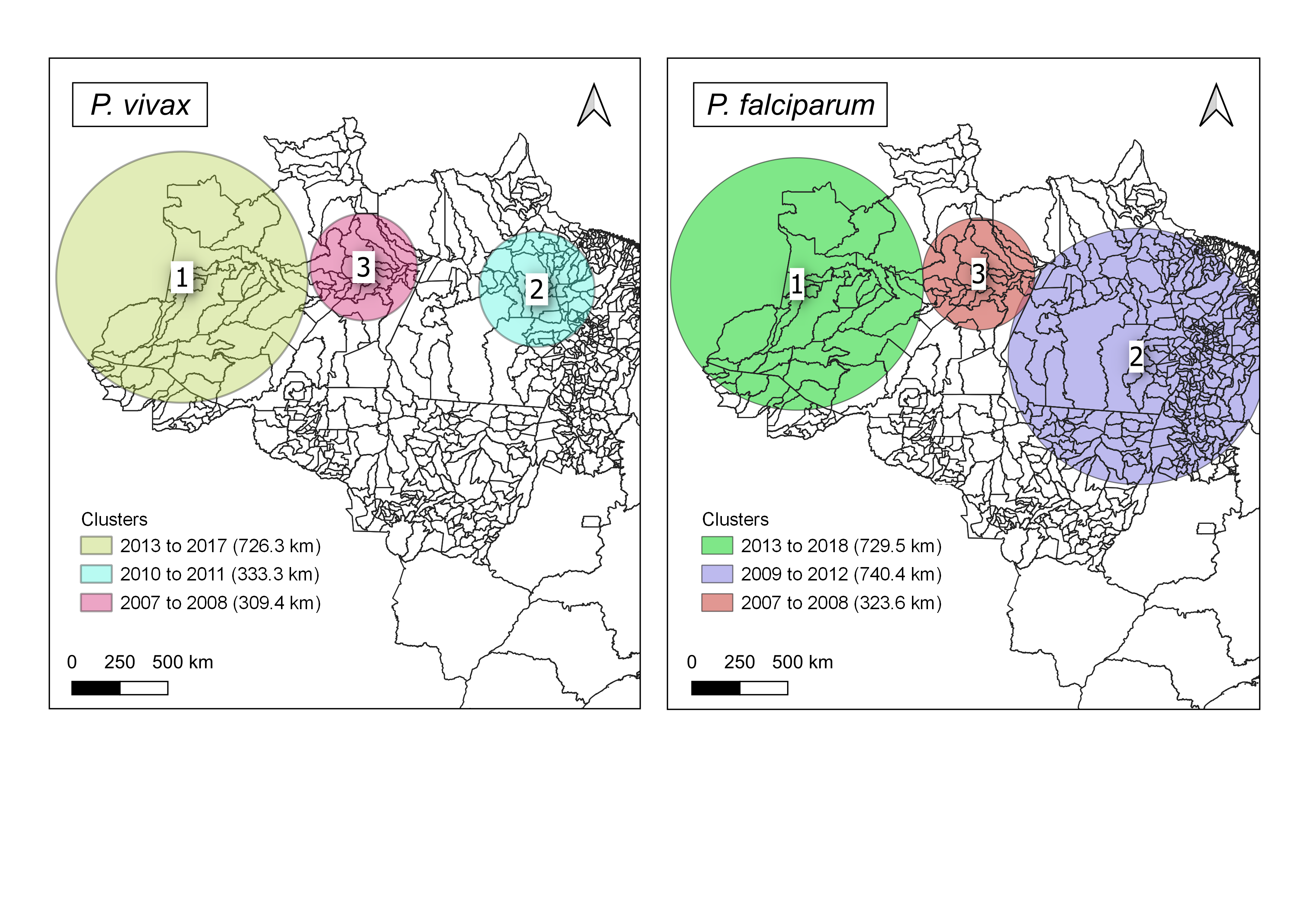

Supplement: Supplementary file 1 [file ijerph-20-06497-s001.zip › Figure S7.png]

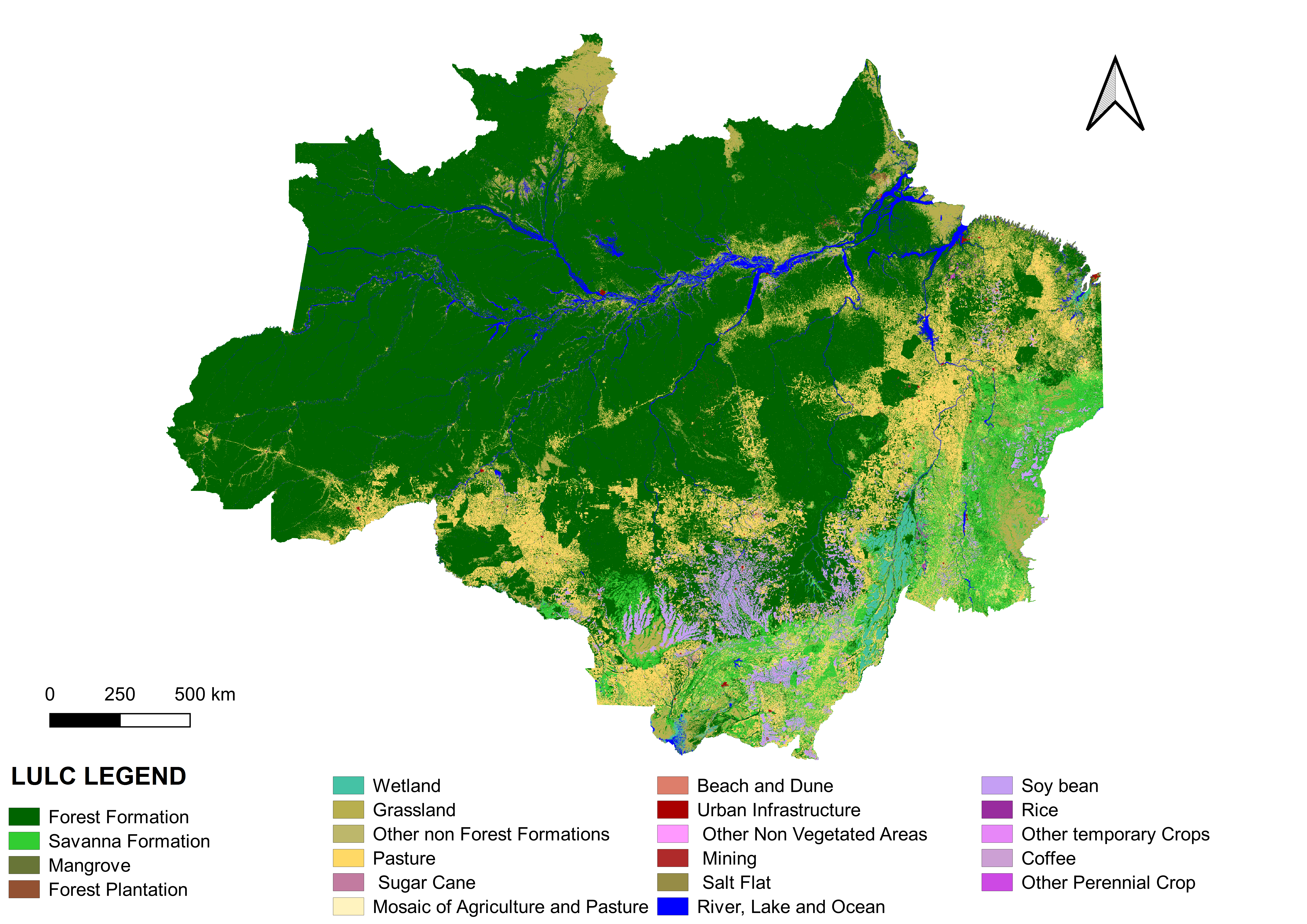

Supplement: Supplementary file 1 [file ijerph-20-06497-s001.zip › Figure S8.png]

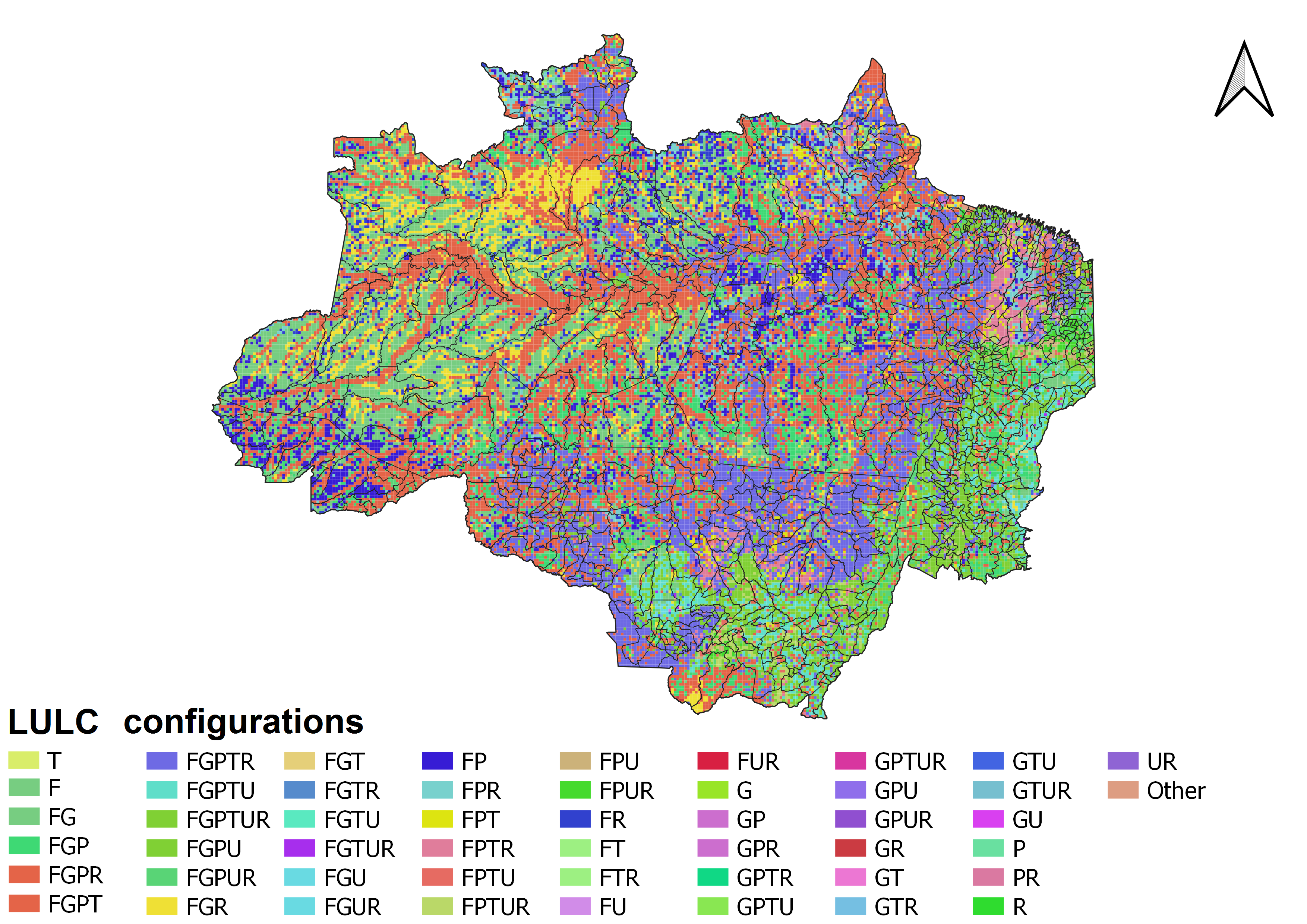

Supplement: Supplementary file 1 [file ijerph-20-06497-s001.zip › Figure S9.png]
